# Revisiting trypanosomatid nucleoside diphosphate kinases

**DOI:** 10.1590/0074-02760210339

**Published:** 2022-02-09

**Authors:** Mariana R Miranda, Melisa Sayé, Chantal Reigada, Facundo Galceran, Marcos Rengifo, Belen J Maciel, Fabio A Digirolamo, Claudio A Pereira

**Affiliations:** 1 Universidad de Buenos Aires, Facultad de Medicina, Instituto de Investigaciones Médicas A Lanari, Buenos Aires, Argentina; 2 Consejo Nacional de Investigaciones Científicas y Técnicas, Universidad de Buenos Aires, Instituto de Investigaciones Médicas, Laboratorio de Parasitología Molecular, Buenos Aires, Argentina

**Keywords:** nucleoside diphosphate kinase, nucleotides, nucleotide metabolism, multifunctionality, drug target

## Abstract

**BACKGROUND:**

An increasing amount of research has led to the positioning of nucleoside diphosphate kinases (NDPK/NDK) as key metabolic enzymes among all organisms. They contribute to the maintenance the intracellular di- and tri- phosphate nucleoside homeostasis, but they also are involved in widely diverse processes such as gene regulation, apoptosis, signal transduction and many other regulatory roles.

**OBJETIVE:**

Examine in depth the NDPKs of trypanosomatid parasites responsible for devastating human diseases (e.g., *Trypanosoma cruzi*, *Trypanosoma brucei* and *Leishmania* spp.) which deserve special attention.

**METHODS:**

The earliest and latest advances in the topic were explored, focusing on trypanosomatid NDPK features, multifunctionality and suitability as molecular drug targets.

**FINDINGS:**

Trypanosomatid NDPKs appear to play functions different from their host counterparts. Evidences indicate that they would perform key roles in the parasite metabolism such as nucleotide homeostasis, drug resistance, DNA damage responses and gene regulation, as well as host-parasite interactions, infection, virulence and immune evasion, placing them as attractive pharmacological targets.

**MAIN CONCLUSIONS:**

NDPKs are very interesting multifunctional enzymes. In the present review, the potential of trypanosomatid NDPKs was highlighted, raising awareness of their value not only with respect to parasite biology but also as molecular targets.

Nucleoside diphosphate kinases (NDPK/NDK, EC 2.7.4.6), are widely conserved enzymes across all organisms involved in several highly important pathways. The main role they fulfil - and the first one to be associated to them -, is the maintenance of intracellular di- and tri-phosphate nucleoside pools through the following reaction:

N1TP + N2DP ↔ N1DP + N2TP

The catalysis process involves the phosphorylation of a conserved histidine residue which in turn phosphorylates the NDP through a ping pong mechanism.^1^

These enzymes are considered multifunctional since different roles have been associated with them, climbing from housekeeping enzymes to gene expression regulators among others. Therefore, they are key metabolic proteins worthy of being studied not only for their biological importance but also as possible drug targets against pathogens.

Trypanosomatids are kinetoplastid organisms belonging to the family Trypanosomatidae*,* which is exclusively composed of protozoan parasites and includes species that cause major diseases in humans.^2^ *Trypanosoma cruzi*, *Trypanosoma brucei* and *Leishmania* spp. are the three best known and more studied trypanosomatids due to their medical importance. These parasites are responsible for Chagas disease or American trypanosomiasis, human African trypansomiasis (HAT) or sleeping sickness and different forms of Leishmaniasis, respectively, affecting millions of people around the world.^2^ The inefficient diagnosis and poor effective treatments, in addition to socioeconomic and cultural factors, make them neglected diseases. Thus, research in this field is challenging and implies an extra effort focused on disease comprehension and development of new therapies. Due to their particular features and participation in high profile pathways, we intend, in this review, to raise awareness of the trypanosomatid NDPKs potential. We pay special attention in discussing what is known to date about isoforms present, moonlighting activity and suitability as drug targets in the three major trypanosomatids, *T. cruzi*, *T. brucei* and *Leishmania* spp.

Importance of NDPKs in the metabolism of nucleotides

According to their catalytic activity, NDPKs participate in the nucleotide metabolism, which is essential for all living organism. Purines and pyrimidines are nucleotide precursors, and nucleotides are the building blocks of DNA and RNA and also act as energy suppliers (i.e., ATP and GTP). Pyrimidines nucleotides (i.e., UTP, CTP, dCTP and TTP) availabilty seems to be exclusivily dependent on NDPKs activity,^3^ ^,^ ^4^ positioning NDPKs in a key role. In constrast to humans, trypanosomatids lack nine of the ten genes responsible for *de novo* purine synthesis and hence they are unable to synthesise their own purines.^5^ ^,^ ^6^ In this regard, purine salvage from the host through their uptake took relevance and several transporters have been characterised.^7^ ^,^ ^8^ These facts constitute an Achilles heel for these parasites and highlight the importance of the NDPKs during the life cycles of these organisms, since they are involved in the process by which free purines and pyrimidines are converted into nucleosides and subsequently into nucleotides.

The NDPK/NME family in trypanosomatids

The NDPK protein family is encoded by NME genes, initially called NM23 (non-metastatic 23) after the first identified member NM23-H1/NME1, which is associated with the tumor metastatic process.^9^ This family includes evolutionarily conserved proteins present in prokaryotes, eukaryotes and also in some viruses.^10^ In vertebrates, the NME/NDPK family is composed of ten proteins (NME1 to 10) which are divided in two groups based on phylogenetic analyses.^11^ ^,^ ^12^ ^,^ ^13^ Group I corresponds to canonical NDPKs NME1 to 4, sharing 58 to 88% identity with each other and all isoforms present NDPK catalytic activity; group II (NME5 to 10) is comprised of more divergent proteins, which share only 25 to 45% identity with group I and between each other.^11^ ^,^ ^12^ Ulloa et al.^14^ were the first to describe NDPK activity in trypanosomatids through purification and biochemical characterisation of a soluble NDPK from epimastigotes of *T. cruzi*. Later, four NDPK isoforms from this parasite were identified. Three of them have been well characterised, named TcNDPK1, with similar characteristics to the enzyme previously described by Ulloa et al.,^14^ TcNDPK2 and TcNDPK3, and the fourth corresponds to a putative isoform, TcNDPK4.^15^ ^,^ ^16^ TcNDPK1, the only one that belongs to group I, is a canonical isoform, localised to the nucleus and cytosol of the parasites. TcNDPK2 and TcNDPK3 are long NDPK isoforms containing N-terminal domains called DM10 and belong to group II. The N-terminal domain of TcNDPK2, is capable of targeting the protein to the cytoskeleton and flagellum of *T. cruzi*,^15^ ^,^ ^17^ ^,^ ^18^ which in addition localised to the cytosol. On the other hand, TcNDPK3 has glycosomal localisation and is predominantly expressed in the mammalian stage trypomastigote.^16^ All *T. cruzi* NDPK isoforms present orthologues in *T. brucei* and *L. major*, with the exception of TcNDPK4 that is not present in *Leishmania* spp.^15^ To date, the *T. brucei* orthologue of TcNDPK1, TbNDPK1, has been the only one characterised in the parasite. This protein is expressed in the bloodstream and procyclic forms and, likewise to TcNDPK1, it is localised predominantly in the cell nucleus.^19^ Besides its cytosolic and nuclear localisation, TcNDPK1 is also present in lipidic vesicles secreted by both epimastigotes and metacyclic trypomastigotes.^20^ Similarly, TbNDPK1 has been found to be secreted by bloodstream trypomastigotes of different *T. brucei gambiense* strains,^21^ while canonical NDPKs from promastigotes of *L. major* and *L. amazonensis* (LmNDKb and LaNDKb respectively, orthologues of TbNDPK1 and TcNDPK1) have been located in microsomal fractions enriched with vesicles that ultimately will go to the cell membrane and/or for secretion.^22^ The figure shows all the mentioned enzymes, integrating their localisation and possible functions, which are discussed in the section below.

**Figure:**
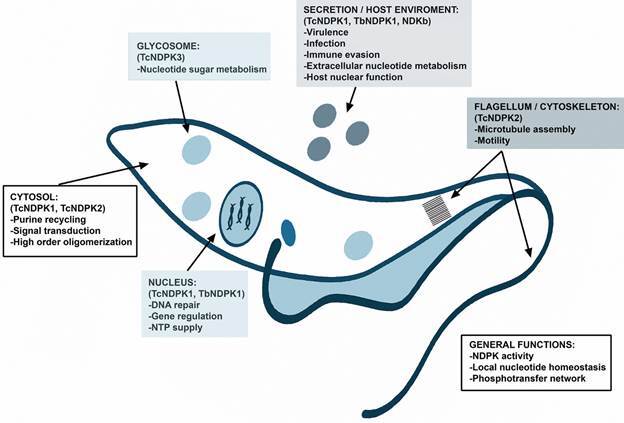
Scheme of a trypanosomatid showing the characterised nucleoside diphosphate kinases (NDPKs), localisation and possible multiple functions.

Several crystal structures of canonical NDPKs from different organisms have been solved, including bacteria, fungi, amoebas, plants, mammals, protist and even viruses.^10^ These determinations revealed different structural assemblies for the NDPKs, which can be found as dimers, tetramers or hexamers. Tetramers are commonly found in prokaryotes while hexamers in eukaryotes. In accordance with this, crystal structures from trypanosomatid NDPKs are available and correspond to hexamers.^23^ ^,^ ^24^ ^,^ ^25^ This quaternary structure is key for proper function of the enzyme. Canonical NDPKs have subunits of about 150 amino acid and present a very similar fold based on the αβ sandwich or ferredoxin fold. Two additional features characterise the structure of the NDPK subunit: the “Kpn-loop” and the C-terminal residues, which complete the fold. The trypanosomatid NDPKs from group I share all these features. For example, TcNDPK1 and TbNDPK1 are formed by 153 amino acids while LmNDKb has a length of 151 amino acids. Furthermore, a mutation in the Pro95 residue (P95S, located in the Kpn-loop) and a deletion in the four carboxy-terminal amino acids of TcNDPK1 not only diminish the NDPK activity but also affect the ability of GFP-fused TcNDPK1 to form granules in the parasite highlighting the relevance of the quaternary structure.^26^ The active site of NDPKs is formed by the nucleotide binding site and the strictly conserved catalytic histidine (H118 in human NME2 and H117 in NDPK1/NDKb from *T. cruzi*, *T. brucei* and *Leishmania* spp.). As expected, replacement of His117 for Asn (H117N) in TcNDPK1 produces an inactive protein, with only about 20% of activity compared to wild-type TcNDPK1.^26^ Similarly, LaNDKb H117A mutant cannot autophosphorylate and shows negligible *in vitro* kinase activity.^25^ The table lists all the trypanosomatid NDPKs identified to date, summarising their main features such as length, characterisation status, localisation and human orthologues.

**TABLE:** The trypanosomatid nucleoside diphosphate kinases (NDPKs)

	Isoform	Length (aa)	Acc. No.	NDPK activity	Subcellular localisation	Secretion	human orthologues^*c*^ (%similarity-identity)	Reference
*T. cruzi*	TcNDPK1	153	TcCLB.508707.200	Yes	Cytosol Nucleus	Lipidic vesicles	NME1/2/3/4 (64.2-53.1/74.0-64.3/67.3-52.6/55.1-43.9)	^(15)^ ^(20)^ ^(35)^
	TcNDPK2	334	TcCLB.508461.400	Yes	Cytoskeleton Flagellum	nd	NME7 (47.7-32.6)	^(15)^ ^(18)^
	TcNDPK3	349	TcCLB.510879.210	Yes	Glycosome	nd	No	^(16)^
	TcNDPK4	632	TcCLB.508989.100	Putative	nd	nd	No	^(15)^
*T. brucei*	TbNDPK1	153	Tb927.11.16130	Yes	Nucleus	Secretome	NME1/2/3/4 (67.6-56.4/77.3-64.9/69.6-54.4/55.6-44.4)	^(19)^ ^(21)^
	TbNDPK2	334	Tb927.9.11260	Putative	nd	nd	NME7 (46.2-32.4)	^(15)^
	TbNDPK3	349	Tb927.4.1720	Putative	nd	nd	No	^(15)^
	TbNDPK4	634	Tb927.8.4510	Putative	nd	nd	No	^(15)^
*L. major* ^*a*^	LmNDPK1 (LmNDKb)	151	LmjF.32.2950	Yes	nd	Microsome^*b*^	NME1/2/3/4 (66.1-54.8/76.3-63.8/68.6-54.4/56.1-43.3)	^(22)^ ^60^
	LmNDPK2	337	LmjF.35.3870	Putative	nd	nd	NME7 (48.3-33.2)	^(15)^
	LmNDPK3	343	LmjF.34.2980	Putative	nd	nd	No	^(15)^

Summary of the main features of the NDPK isoforms of *Trypanosoma cruzi*, *Trypanosoma brucei* and *Leishmania major* (column 1) characterised to date: gene name (column 2), amino acid length (column 3), accession numbers from GeneDB (column 4), NDPK activity (active or putative, column 5), subcellular localisation (column 6) and NDPK secretion by the parasites (column 7). nd: not determined; *a*: NDPKs have also been described in *Leishmania amazonensis* and *Leishmania braziliensis* (LaNDKb and LbNDPKb); *b*: it has also been demonstrated in *L. amazonensis*;^22^ *c*: OrthoMCL DB (https://www.orthomcl.org) was used for human orthologous identification, percentages of similarity and identity were calculated with the Vector NTI v11 software (Invitrogen).

Trypanosomatid NDPKs as enzymes participating in multiple processes

Far from being housekeeping enzymes, the accumulated evidence implicates NDPKs in very different cellular processes, not fully understood until now. Although the NDPK family is composed of canonical and divergent NDPKs, multifunctionality is best known for canonical isoforms because these are ubiquitous, highly conserved and hence, more studied. As a result, a wide variety of unrelated processes from prokaryotes and eukaryotes have been reported in which canonical enzymes play a role. These processes include the following: (a) signal transduction,^27^ ^,^ ^28^ (b) virulence and host-pathogen interactions,^29^ ^,^ ^30^ ^,^ ^31^ ^,^ ^32^ (c) apoptosis,^33^ (d) metastasis,^9^ (e) gene regulation^34^ and (f) genome integrity,^35^ ^,^ ^36^ ^,^ ^37^ among others.^38^ ^,^ ^39^ ^,^ ^40^ One intriguing role that deserves special attention, is the case of human NME2, first discovered as Puf, which acts as a transcription factor regulating c-myc expression through binding to a G-quadruplex DNA secondary structure within the promoter region.^34^ On the other hand, divergent NDPKs are also associated to functions different from the housekeeping one, mainly related to their local cellular distribution. For example, NDPKs bound to tubulin/cytoskeleton are believed to be involved in microtubule assembly and motility.^38^ The so-called multifunctionality can be controversial as many of the various processes associated with these enzymes probably derive from their kinase activity. However, independent kinase roles (e.g., nuclease activity or transcription regulation) have also been reported, expanding their functions and allowing them to be considered moonlighting proteins.

Trypanosomatid NDPKs are also multifunctional enzymes. In a first report the presence of multiple isoforms were identified in these organisms and, initially, a role in cellular energetics was postulated^15^ in the so-called “phosphotransfer networks”.^41^ Later, isoform localisation was confirmed to be in the cytosol, nucleus, glycosomes, cytoskeleton and flagella in *T. cruzi*, as mentioned above, and thus being distributed all along the parasite body.^15^ Within the different NDPK isoforms, functions of the sole canonical ones of *T. cruzi* and *Leishmania* spp. were studied a little more. Although it is not a physiological role, they are involved in antiparasitic drug resistance. NDPK from *L. braziliensis*, LbNDKb, is phosphorylated and overexpressed in an antimony resistant line and similarly TcNDPK1 is overexpressed in benznidazole resistance phenotypes^42^ ^,^ ^43^ not only in *in vivo* resistant clones but also in *in vitro* induced-resistant clones. This report is in agreement with preliminary results where *T. cruzi* epimastigotes overexpressing TcNDPK1 presented more tolerance to increasing concentrations of benznidazole than wild-type parasites (unpublished data). One final effect of antiparasitics is the promotion of DNA damage, which was established for benznidazole in *T. brucei*.^44^ Furthermore, NDPKs seem to be associated with nucleic acids-related functions like gene regulation and DNA repair machinery, in addition to the intrinsic nuclease activity present in canonical enzymes. In this regard, nuclease activity was confirmed for TcNDPK1^17^ and, moreover, in a recent study, it was demonstrated that TcNDPK1 is involved in *T. cruzi* genome integrity maintenance, exerting a protective role against genotoxic agents probably through the up-regulation of enzymes involved in DNA repair.^35^ Therefore, the benznidazole resistant phenotype could be associated with this TcNDPK1 function.

Another particular trait is that the secretion of these enzymes opens new insights on functions related to virulence and parasite-host interactions. Delivery of NDPKs inside the host and even intracellularly might trigger different pathways to coordinate infection as has been reported for other pathogens.^31^ Despite they do not possess any typical secretion signal, a vesicle-mediated secretion pathway was evidenced for TcNDPK1 since it was enriched in the vesicle fraction obtained from epimastigotes, as well as for metacyclic trypomastigotes, the stage of the parasite relevant in the infection process.^20^ It is known that extracellular ATP constitutes a danger signal that activates the immune response during infection and several ATP consuming enzymes are secreted in order to interfere with this process.^45^ Thus, released NDPKs could contribute to parasite infection in this way. Supporting this hypothesis, it was demonstrated that secreted LaNDKb is involved in preserving the integrity of the host cell in benefit of the parasite, since purified or recombinant NDKb decreases extracellular ATP preventing macrophages lysis.^32^

Functions associated to the oligomerisation degree were also proposed. Even though hexamers are known as active oligomers, *in vivo* granules were observed for human and trypanosomatid canonical NDPKs. In *T. cruzi* it was discovered that TcNDPK1 forms large granules that depend on the hexamer assembling and are present in some stress conditions such as starvation and metacyclogenesis.^26^ These precedents suggest that some other novel functions could be associated to a higher order of oligomerisation as for example, signalling, substrates sequestration, regulation or buffering of enzymatic activity, etc.^46^ Likewise, glycosomal isoforms could be related to unusual functions associated to this particular organelle,^16^ as for example, nucleotide sugar biosynthesis, which was recently reported to occur in the glycosomes of *T. brucei*, unlike other eukaryotes.^47^ Also, glycosomes are peroxisomes related organelles and NDPKs have been involved in the division of the latter.^39^

The figure represents all functions trypanosomatid NDPKs could perform, some experimentally confirmed in the parasites and some inferred from the reported evidences among organisms.

Trypanosomatid NDPKs as molecular targets

Although the trypanosomatid NDPKs have not been validated as drug targets yet, there are many examples in other organisms demonstrating that they are essential or conditionally essential proteins. One interesting and well known example is the “killer of prune” (Kpn) mutation in the *ADW* gene (*NME1*/*2* homologues) of *Drosophila melanogaster*. A single amino acid change (Ser97Pro) produces a dominant lethal phenotype when it is combined with the *prune* mutation.^48^ In mammals, NME1 and NME2 double knockout mice present developmental failures and die immediately after birth.^49^ In the filamentous fungus *Aspergillus flavus*, NDPKs are involved in spore development and sclerotia production affecting the fungus survival structures^50^ and, interestingly, in *A. nidulans* SwoHp NDPK is an essential protein.^51^ Related to human protozoan parasites, in apicomplexa, silencing of NDPK strongly inhibited *Cryptosporidium parvum* proliferation in human intestinal cells.^52^

The importance of NDPKs in the metabolism of nucleotides and their participation in multiple processes make them attractive targets since disrupting a single enzyme will affect several cellular functions simultaneously. As was mentioned, an interesting feature of trypanosomatids is the absence of metabolic pathways for *de novo* synthesis of purines,^6^ which makes nucleotide metabolism a weakness for the parasites and where NDPKs fulfil a central role. Also, the presence of an isoform in the glycosomes, constitutes an additional promising feature, not only because glycosomes are organelles exclusive of trypanosomatid organisms, but also because glycosomal isoforms could be associated to unusual unknown functions and no human counterpart exists.^16^ Therefore, an ideal approach against these parasites could be a combinatory therapy disrupting both, purine transport and NDPKs simultaneously.

Stanly Paul et al.^53^ carried out an *in silico* gene knockout study to rank predicted drug targets in *L. major* where NDKb was one of the selected genes. Other authors used different criteria to prioritise putative drug targets that include essentiality, druggability range, assayability, presence of experimental crystal structure and being different from human proteins.^53^ Although the last requirement is not strictly fulfilled when talking about canonical NDPKs, significant differences have been proven in the electronegative potential between human and trypanosomatid NDPKs that are valuable features for specific ligand design. This point is particularly worthy because NDPK activity is lost not only when the catalytic site is disturbed but also when hexamers are disassembled.^54^ After this preliminary work, two drug discovery studies were carried out in *Leishmania* spp using NDKb as a target. Based on the NDKb structure Mishra et al.^25^ identified five enzyme inhibitors and one of them (BTB13319) is also active against promastigotes at low micromolar concentration. In addition, Vieira et al.^55^ found a pyrrole-indolinone, a Sunitinib (antineoplastic) analogue, that inhibits NDKb binding active site in a similar conformation to natural nucleotides. This compound has similar efficacy as amphotericin B against some *Leishmania* species. Despite the low leishmanicidal activity of both compounds, they provide promising scaffolds for rational drug design targeting trypanosomatid NDPKs.

Even though no strong and specific inhibitor of NDPK activity has been identified so far, early studies reveal interest in finding effective NDPK inhibitors. For example, in 1972 it was published that the antibiotic cyclamidomycin (desdanine) inhibits *Escherichia coli* NDK.^56^ Then, several anionic dyes were also reported to bind to the active site of NDPK including the antiallergic drug chromoglycate, the iodinated xanthene Rose Bengal and the chromophores Cibacron Blue and Procion Blue.^54^ In addition, NDPKs were studied for antiviral nucleotide analogues activation in order to find new therapies for AIDS.^57^ ^,^ ^58^ All these evidences encourage the search for novel trypanocidal compounds targeting NDPKs, for example, through computer-assisted techniques as the currently used methodology of drug repurposing.^59^

Concluding remarks

NDPKs are intriguing enzymes. It seems that they are involved in every process you are looking for. Their moonlighting activity or multifunctionality is a very interesting topic that deserves to be investigated. Here, we present a complete and summarised overview of the trypanosomatid NDPKs which meet additional importance due to the organisms they belong to. The trypanosomatids we reviewed here are human’s parasites, with complex life cycles composed of host- intracellular and extracellular stages, where NDPKs could play key roles not only in the parasite metabolism (i.e., nucleotide homeostasis, drug resistance, DNA damage responses, gene regulation, etc.), but also in host-parasite interactions, infection, virulence and immune evasion. In accordance with their relevant roles and the singular features that trypanosomatid NDPKs have (i.e., different from host ones, not only in biochemical features but also in processes they participate), they are under the spotlight of several studies concerning the discovery of drug targets and development of new strategies for the treatment of such neglected diseases caused by these pathogens. Therefore, two questions arise from the presented scenario, one related to metabolic issues and the other concerning therapeutics: Are NDPKs master enzymes involved in the overall management of the parasite own cell and that of the host? Are trypanosomatid NDPKs promising drug targets to combat the parasites? Investigations are going that way.
